# Integrin alpha9 emerges as a key therapeutic target to reduce metastasis in rhabdomyosarcoma and neuroblastoma

**DOI:** 10.1007/s00018-022-04557-y

**Published:** 2022-10-11

**Authors:** Natalia Navarro, Carla Molist, Júlia Sansa-Girona, Patricia Zarzosa, Gabriel Gallo-Oller, Guillem Pons, Ainara Magdaleno, Gabriela Guillén, Raquel Hladun, Marta Garrido, Miguel F. Segura, Lourdes Hontecillas-Prieto, Enrique de Álava, Berta Ponsati, Jimena Fernández-Carneado, Ana Almazán-Moga, Mariona Vallès-Miret, Josep Farrera-Sinfreu, Josep Sánchez de Toledo, Lucas Moreno, Soledad Gallego, Josep Roma

**Affiliations:** 1 Laboratory of Translational Research in Child and Adolescent Cancer, Vall d’Hebron Research Institute (VHIR), Hospital Universitari Vall d’Hebron, Universitat Autònoma de Barcelona, Bellaterra, Spain; 2 Pediatric Oncology and Hematology Department, Hospital Universitari Vall d’Hebron, Universitat Autònoma de Barcelona, Bellaterra, Spain; 3 Pediatric Surgery Department, Hospital Universitari Vall d’Hebron, Universitat Autònoma de Barcelona, Bellaterra, Spain; 4 Pathology Department, Hospital Universitari Vall d’Hebron, Universitat Autònoma de Barcelona, Bellaterra, Spain; 5 Institute of Biomedicine of Seville (IBiS), Hospital Universitario Virgen del Rocío/CSIC, University of Seville/CIBERONC, Seville, Spain; 6 Department of Normal and Pathological Cytology and Histology, School of Medicine, University of Seville, Seville, Spain; 7 BCN Peptides, Pol. Ind. Els Vinyets Els Fogars II, Sant Quintí de Mediona, Barcelona, Spain

**Keywords:** Cancer, Paediatric cancer, Solid tumours, Dissemination, Progression

## Abstract

The majority of current cancer therapies are aimed at reducing tumour growth, but there is lack of viable pharmacological options to reduce the formation of metastasis. This is a paradox, since more than 90% of cancer deaths are attributable to metastatic progression. Integrin alpha9 (ITGA9) has been previously described as playing an essential role in metastasis; however, little is known about the mechanism that links this protein to this process, being one of the less studied integrins. We have now deciphered the importance of ITGA9 in metastasis and provide evidence demonstrating its essentiality for metastatic dissemination in rhabdomyosarcoma and neuroblastoma. However, the most translational advance of this study is to reveal, for the first time, the possibility of reducing metastasis by pharmacological inhibition of ITGA9 with a synthetic peptide simulating a key interaction domain of ADAM proteins, in experimental metastasis models, not only in childhood cancers but also in a breast cancer model.

**Supplementary Information:**

The online version contains supplementary material available at 10.1007/s00018-022-04557-y.

## Introduction

Metastasis is the main cause of cancer-associated mortality and one of the most indicative prognostic factors for cancer patients [1–4]. This highlights the necessity to better understand the biology of this process and to develop new therapeutic approaches against it to reduce cancer mortality. Metastasis requires that tumour cells successfully overcome a series of obstacles in order to leave the primary tumour and form a secondary tumour in the “soil” tissue [4]. Many proteins are involved in the complex regulation of this process, of particular note is the family of integrins. Altered integrin expression has been linked to many cancers, and integrins, or their downstream signalling proteins, have been related to almost all the different phases of the metastatic cascade [5–7]. Integrins are a family of transmembrane receptors which mediate cell adhesion to the extracellular matrix (ECM) and participate in cytoskeleton organization. Although integrins do not have intrinsic enzymatic activity, they can generate bidirectional signals (outside-in and inside-out) as they can interact with ECM ligands, cell surface proteins, intracellular adaptor proteins, such as talin, and other signalling molecules, such as focal adhesion kinase (FAK) and the Src family kinases. These bidirectional signals can regulate cellular behaviour including survival, proliferation, migration and ECM assembly in response to the cellular environment [8, 9].

The Integrin alpha9 (ITGA9) protein is composed of the α9 itself and a β1 subunit. Unlike other integrins, ITGA9 does not depend on the Arg-Gly-Asp (RGD) sequence for ligand recognition [10] and binds to many different ligands (such as fibronectin, ADAMs, tenascin-C and L1CAM, among many others), some of which are in common with Integrin alpha4 (ITGA4) [11]. ITGA9 has been related to cell migration, cell invasion and epithelial–mesenchymal transition (EMT) of tumour cells [12–15], all important steps for metastatic colonization. The previous results from our laboratory showed ITGA9 as a Notch pathway target and as an interesting player in the metastatic potential of rhabdomyosarcoma cells, whose inhibition by miR-7 and miR-234-5p exerted strong anti-oncogenic effects [13, 14]. Besides, ITGA9 has been strongly associated with patient outcomes in other tumour types. ITGA9 has been related to high tumour grade, presence of distant metastases, and reduced overall survival in breast cancer patients [16, 17]. In the case of glioma, ITGA9 expression correlated with disease grade: normal tissue showed no expression of ITGA9 in astrocytes, oligodendrocytes or neurons, whereas glioblastoma multiforme and giant-cell glioblastoma showed high expression of this protein [18]. In melanoma, and under the regulation of several non-coding RNAs and miRNAs, ITGA9 has been related to the oncogenic regulation of proliferation, apoptosis and metastasis [19, 20].

One of the most remarkable families of ITGA9 interactors is the ADAM family, which has some members highly expressed in many types of cancer and can regulate cell–cell adhesion or cell-ECM interaction through its binding to adhesion molecules such as integrins and syndecans [21]. Overall, overexpression of several ADAM proteins has been correlated with metastasis and poor survival in a wide range of cancers [10]. Difficulties in the study of metastasis, such as the lack of in vitro assays for the whole process, or selection of advanced stage patients to enter clinical trials, have hindered the translation of anti-metastatic agents with promising preclinical results into the clinic [22]. Herein, we describe the anti-metastatic effects of the genetic inhibition of ITGA9 in two different cancers: rhabdomyosarcoma (RMS), the most prevalent soft tissue sarcoma in children [23], and neuroblastoma (NB), the most common extracranial solid tumour in children [24]. Subsequently, its pharmacological inhibition was also tested in these two types of cancer, but also in breast cancer (BC), the most frequent cancer in women [2]. These three cancers have a different cell of origin and affect different tissues and populations. Nevertheless, they have one trait in common: although they have a good general overall survival (around 70% for RMS and NB [25, 26], and around 80% for BC [27]), a subset of patients develop metastasis and this drastically worsens their survival rates (approximately 30% for RMS, 50% for NB [28] and below 40% for BC patients [27]). Here we propose ITGA9 as a key player in the metastatic spreading of these tumours and introduce a new anti-metastatic peptide (inspired by a key domain frequently found in the ADAM family of proteins with the aim of inhibiting ITGA9 activity), with very promising preclinical evidence on preventing the establishment of metastasis, which may have potential applicability in a broad spectrum of tumour types.

## Materials and methods

### Cell culture

RMS cell lines RH30 and RD were cultured in Minimum Essential Medium with Earle’s Salts (Biowest), NB cell lines BE(2)-C and CHLA90 were grown in Iscove’s Modified Dulbecco’s Medium (Thermo Fisher Scientific) and BC cell lines MDA-MB-231 and MDA-MB-468 were cultured in Dulbecco’s Modified Eagle Medium (Thermo Fisher Scientific). All media were supplemented with 10% FBS (Sigma), 1 × non-essential amino acids, 100U/ml penicillin and 0.1 mg/ml streptomycin (all from Biowest). All cell lines were maintained at 37 °C in a controlled atmosphere of 5% CO_2_ and were periodically tested for mycoplasma by PCR and authenticated by short tandem repeat profiling.

### Generation of cell lines with stable shRNA expression and rescue experiments

Genetic ITGA9 downregulation was performed by shRNA cloned into the lentiviral vector pGIPZ (GE Healthcare Dharmacon, clone ID: V3LHS_388097 and V3LHS_388100). Lentiviruses were produced in HEK293T cells and 2 × 10^5^ target cells were seeded in 60 mm dishes and incubated together overnight. Positively transduced cells were selected with puromycin (1ug/ml, Sigma-Aldrich). Four different shRNAs were tested and the ones that reduced ITGA9 expression more efficiently were selected for further experiments. Rescue experiments to overexpress ITGA9 were performed using pcDNA3.1(+) vector (GenScript Biotech), carrying ITGA9 complete cDNA (NM_002207.3). Cells were transiently transfected with 2 μg of each construct using Lipofectamine (Thermo Fisher) and protein analyses were performed after 24 h.

### Western blotting

At 80%-confluence, cells were washed with PBS and scraped in RIPA protein lysis buffer (Thermo Fisher Scientific) supplemented with protease (Roche) and phosphatase inhibitors (Sigma-Aldrich). Cell lysates were incubated for 5 min at 95 °C and after centrifugation at 13000 rpm for 15 min at 4 °C, cell debris was discarded. Supernatant protein concentration was quantified by the DC Protein Assay (Bio-Rad) according to manufacturer’s instructions, and 20-40 µg of total protein was separated on 8–12% SDS-PAGE gels and transferred onto PVDF membranes (GE Healthcare). After blocking with 5% BSA (Sigma-Aldrich) for 1 h, membranes were incubated overnight at 4 °C with the following primary antibodies: anti-ITGA9 (diluted 1:1000, Abnova, H00003680-M01), anti-FAK (diluted 1:1000, Cell Signalling, 3285), anti-pFAK at Tyr397 (diluted 1:500, Cell Signalling, 3283) and anti-actin (diluted 1:10,000, Santa Cruz Biotechnology, sc-1616). Finally, membranes were incubated with the corresponding HRP-conjugated secondary antibodies for 1 h at room temperature and immunoreactive bands were visualized with ECL Prime reagent (GE Healthcare).

### Cell invasion assay

1 × 10^5^ cells resuspended in serum-free medium were seeded in the upper chamber of a Matrigel-coated Transwell (Corning), and the lower chamber was filled with serum-supplemented medium. Cells were incubated at 37 °C for 24 h and fixed with 4% paraformaldehyde (Sigma-Aldrich). After removal of the Matrigel, the cells that migrated to the lower surface of the membrane were stained with Hoechst-33342 (5 ng/ml, Sigma-Aldrich) and were counted under the microscope. Three independent wells were counted for each condition. To assess RA08’s effects on cell invasion (and RGD peptide), cells were previously treated for 48 h with different concentrations (0 nM, 100 nM, 200 nM, 500 nM and 1000 nM), which were also present in the medium during the cell invasion assay.

### Cell proliferation assay

Cell proliferation was measured using crystal violet staining. Cells were seeded at low-density in 96-well plates, and a different initial number of cells were used for each cell line to ensure lineal growth throughout the assay. Media was changed every 48 h, including RA08 (or RGD peptide) treatment (0 nM, 500 nM and 1000 nM). Cells were allowed to proliferate for 5 days and were washed with PBS, stained with 0.5% crystal violet (Sigma-Aldrich) and washed thoroughly with distilled water. Plates were dried overnight and crystals were dissolved with 15% acetic acid (Carl Roth). Finally, absorbance was measured at 590 nm using an Epoch microplate spectrophotometer (Biotek).

### Non-adherent cell growth assay

Anchorage-independent cell growth was measured by WST-1 (Sigma-Aldrich), following the manufacturer’s instructions. Cells were seeded at low density in polyHEMA-coated dishes (Santa Cruz Biotechnology) that prevented cell attachment to the plate and were allowed to grow for 48 h. At the end of the assay, cell spheres were disaggregated with accutase (Fisher Scientific) and WST-1 reagent was added to the medium. Three hours later, absorbance was measured at 440 and 690 nm using an Epoch microplate spectrophotometer (Biotek).

### In vivo experimental tail vein metastasis models

Tumour cells were intravenously injected through the tail vein of five-week-old SCID-beige mice (Charles River Laboratories) housed in specific pathogen-free conditions. Cell number was adjusted according to the cell line’s ability to generate metastases (2 × 10^6^ RD cells, 1.25 × 10^5^ MDA-MB-468 cells, and 1 × 10^4^ and 2 × 10^4^ BE(2)-C cells for the shRNA and RA08 models, respectively). To test RA08 effects, mice were split into three groups of 10 mice each and treated with the vehicle (saline solution) or two different RA08 doses by subcutaneous injection. The first RA08 administration was performed two hours before tail vein injection and then three times per week throughout the experiment. Mice were monitored and their body weight measured twice per week. Ethical endpoint criteria were based on: acute weight loss (> 10% of total body weight), clear detection of metastases (lumps or swelling) or poor general appearance of the animal. Metastatic foci were found in post-mortem analysis of mice fulfilling at least one of these end-point criteria. After euthanasia, all organs were assessed and macroscopic metastases were detected. Then, tissue samples were collected, fixed in formalin (Sigma-Aldrich) and paraffin-embedded. In the case of RMS models, most of the metastases were found in lungs, ovary and suprarenal gland, while in the NB models the majority of metastases were hepatic. For the BC model, lung sections were deparaffinized and rehydrated for incubation with anti-GATA3 antibody (Sigma-Aldrich, L50-823). All procedures were approved by the Ethics Committee of Animal Experimentation of the Vall d’Hebron Research Institute (CEEA) and were in line with EU directive 2010/63/EU.

### Alpha integrin family mRNA expression analyses

Analysis of expression data from patients was performed on publicly available data sets from the R2: Genomic Analysis and Visualization Platform. Davicioni et al. 2006 “Tumor Rhabdomyosarcoma—Davicioni—147—MAS5.0—u133a” [29] data set was used for RMS patients, while Su et al. 2014 “Tumor Neuroblastoma—SEQC—498—RPM—seqcnb1” [30] and Asgharzadeh et al. 2006 “Tumor Neuroblastoma non *MYCN* amplified—Seeger—102—MAS5.0—u133a” [31] were used for NB patients and metastatic non-*MYCN*-amplified subset of patients, respectively.

### Peptide synthesis

RA08 was synthesized by solid-phase peptide synthesis following the Fmoc/tBu strategy with 4-Methylbenzhydrylamine (p-MBHA) resin at the 30 mmol scale. Fmoc-AM-OH linker (2 eq.), HOBT (2 eq.) and DIPCDI (2 eq.) were used for attaching the linker to the resin. Then, synthesis proceeded with the removal of the Fmoc group by treatment with a solution of 20% piperidine in DMF (twice for 5 min and twice for 10 min). The peptide-resin was washed 5 times with DMF, filtered and the amino acids were coupled. Couplings were performed by using Fmoc-AA-OH:DIPCDI:HOBt (3 eq: 3 eq: 3 eq) in DMF (Fmoc-Msa-OH was coupled using 1.5 eq). All these coupling reactions were left to react for 40–60 min. The completion of each coupling reaction was controlled with a ninhydrin test (or a Chloranil test). After completion of the peptide sequence, the peptidyl-resin was washed with DMF, MeOH and Et2O and dried. 92.6 g of peptidyl-resin were obtained. Then, the peptidyl-resin was left to react for 2 h in a TFA mixture (TFA:TIO:TIS:H2O (85:5:5:5) (1:10, w:v)) at room temperature to cleave the peptide from the resin. The resin was washed with TFA and Et_2_O. All the filtrates were plunged into cold ether. The suspension obtained was filtered through a filter plate and the filters discarded. The solid was freeze dried and the peptide crude product obtained (48.5 g) was purified in a semipreparative system equipped with a NW50 Merck column filled with 10 µm of kromasil silica, and the pure fractions analysed in an analytical RP-HPLC were lyophilized. The ion-exchange step was carried out to obtain the peptide with the acetate counterion. The final acetate compound was recovered via filtering and then lyophilized. (13.4 g were obtained.) RA08 was characterized by mass spectrometry by ESI–MS spectrometry (MW = 1806 a.m.u.).

### RA08/ITGA9 binding analysis

The binding of RA08 to ITGA9 was assessed using the method of the cellular thermal shift assay (CETSA) as previously described [32]. Briefly, cells (in the presence or absence of RA08) were exposed to a temperature gradient (from 37 to 75 °C during 3 min). Then, protein extracts were resolved in 3 independent western blots to measure the differences of ITGA9 degradation in the presence or absence of RA08.

In silico analysis of RA08 binding to ITGA9 was performed using the platform *LZerD protein docking suite *[33] to predict the most probable RA08/ITGA9 complex docking structure. The model with the best score was selected to run protein binding energy prediction PRODIGY web server [34] and calculate the binding affinity (in Kcal mol^−1^). The ADAM-12/ITGA9 binding affinity was also calculated with the same web server. All analyses were performed from protein structure predicted by homology-modelling server SWISS-MODEL [35].

### Statistical analysis

At least three independent replicates were performed for each experiment and analysis was performed using GraphPad Prism Software. Error bars in graphical data represent mean ± SD. Statistical significance was determined by Student’s *t* test or one-way ANOVA (when more than two data sets were analysed), considering *p < 0.05, **p < 0.01 and ***p < 0.001. Log-rank test was used for survival analysis.

## Results

### shRNA-mediated ITGA9 downregulation reduced focal adhesion kinase (FAK) phosphorylation

**Fig. 1:**
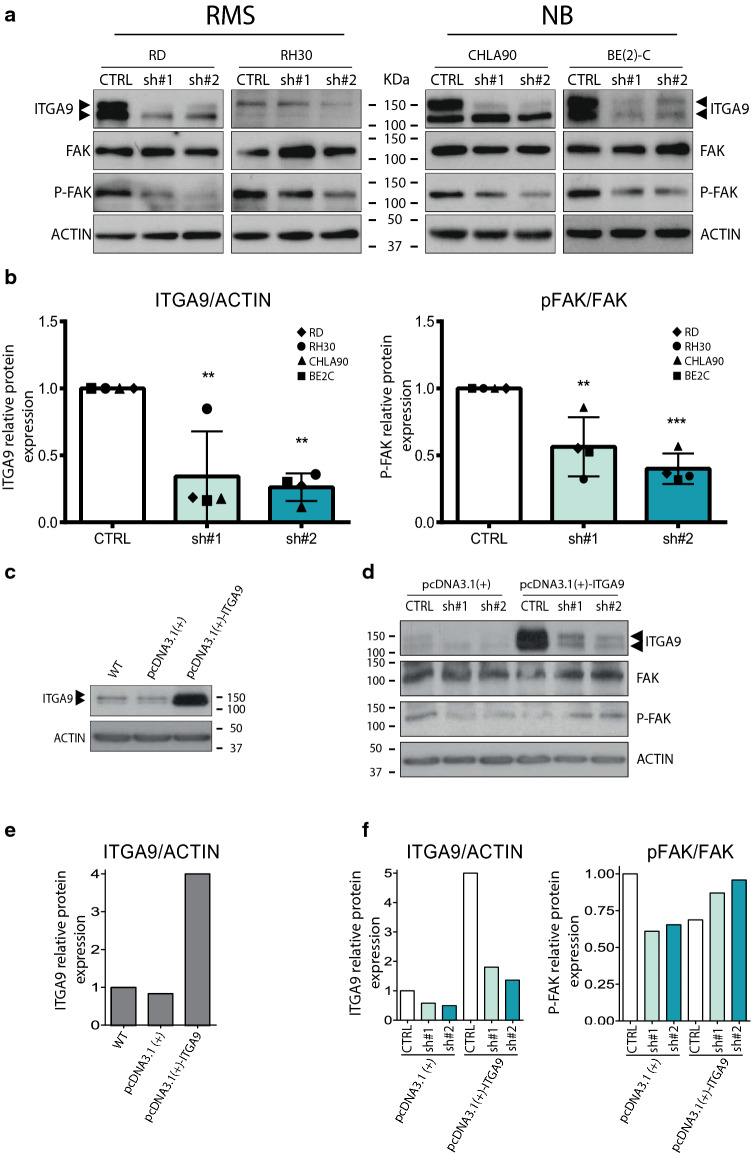

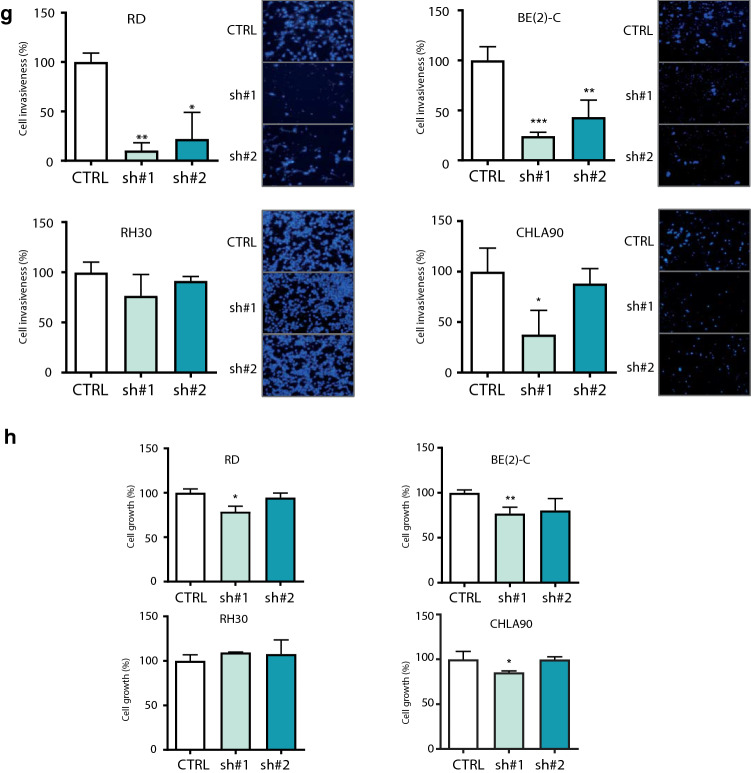
ITGA9 knockdown reduces FAK phosphorylation and cell invasion. **a** Representative immunoblot showing ITGA9 downregulation by two shRNAs. Total-FAK and subsequent reduction of phosphorylated-FAK (at Tyr397) are also shown. **b** Densitometric analysis of western blot from (shown in a), showing ITGA9 levels and the ratio PFAK/FAK. Mean values were derived from single experiments performed in 4 different cell lines**.** Statistical significance (*p < 0.05, **p < 0.01, ***p < 0.001) was obtained after one-way ANOVA test. **c** Western blot to test the capability of the system to overexpress ITGA9 in cells non-transfected (WT), transfected with vector alone (pcDNA3.1(+) and vector with ITGA9 (pcDNA3.1( +)-ITGA9). **d** Rescue experiment showing levels of ITGA9, FAK and P-FAK in all the conditions of the assay (CTRL, sh#1 and sh#2) in the presence or absence of ITGA9 overexpression.** e** and **f** Densitometric analysis of Western blots (shown in c and d, respectively). **g** Relative cell invasiveness after ITGA9 downregulation and representative images of the Transwell-invasion assays. **h** Relative cell proliferation after ITGA9 depletion. All values are expressed in percentage and referred to those of the control condition (for g and h, statistical significance (*p < 0.05, **p < 0.01, ***p < 0.001) was obtained after one-way ANOVA test)

ITGA9 expression was downregulated in cells stably transduced with two specific shRNAs (sh#1 and sh#2) both very effective in reducing the amount of protein compared with empty vector-transduced cells (CTRL). Western blots (Fig. 1a) were carried out to assess the effects of ITGA9 inhibition on FAK expression and phosphorylation (at Tyr397) in RMS and NB cells. The results (Fig. 1a and b) showed a remarkable reduction in FAK phosphorylation in cells with genetic downregulation of ITGA9, whereas total FAK levels were not altered. Interestingly, restoration of ITGA9 expression (similar to control levels) produced a rescue of FAK phosphorylation in the clones infected with shRNA (Fig. 1c–f). Surprisingly, this restoration of FAK phosphorylation was not observed in the control clone (with extremely high ITGA9 overexpression), thereby suggesting that the possible induction of FAK phosphorylation by ITGA9 would be strongly dose-dependent.

### ITGA9 knockdown reduced cell invasion without altering cell proliferation

Cell invasiveness was tested in indicated cell lines transduced with empty vector or shRNAs against ITGA9 (Fig. 1g). The results revealed a considerable reduction in cell invasiveness for the two shRNAs selected in the cell lines RD and BE(2)-C, with a remarkable reduction in sh#1-transduced cells (higher than 75%) and sh#2-transduced cells (higher than 50%). Conversely, in the cell line CHLA90, even though ITGA9 knockdown was not complete, sh#1 produced—albeit moderate—a significant reduction, while in cell line RH30, which expressed low ITGA9 levels (Supplementary Fig. 1a), no significant effect was observed. This finding indicated a crucial role of ITGA9 in cell invasiveness, in a strongly cell line-specific manner. Finally, the shRNA-mediated ITGA9 silencing showed no remarkable effects on proliferation, with just a very slight decrease in RD, BE(2)-C and CHLA90 cell lines (solely observed with sh#1) (Fig. 1h).

### The impairment of ITGA9 expression promoted a decrease in RD and BE(2)-C cell lines of both in vitro non-adherent cell growth and in vivo metastasis experimental models

**Fig. 2:**
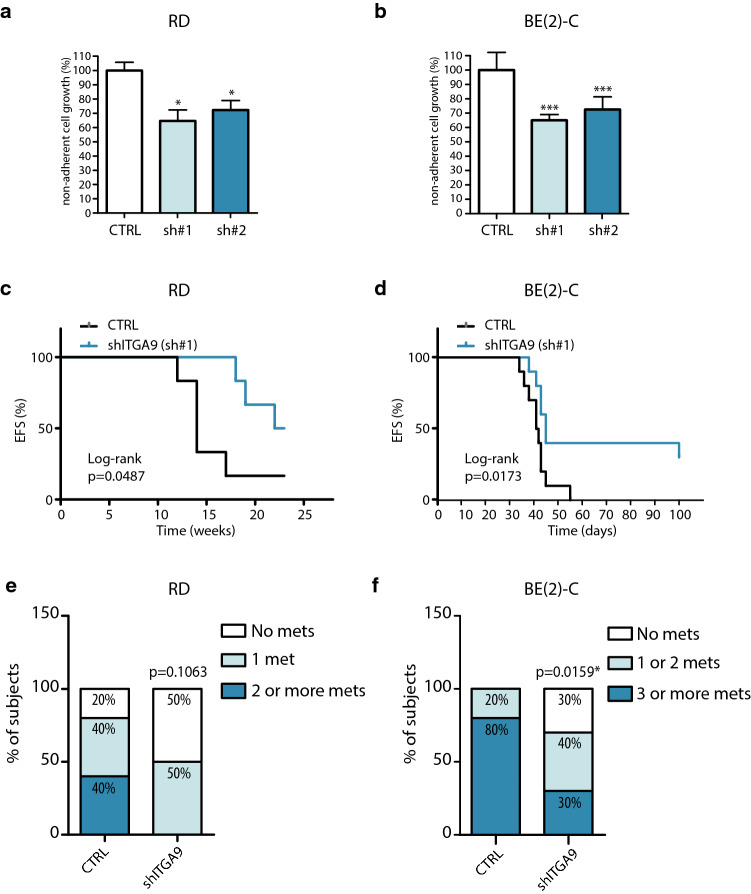
ITGA9 knockdown decreases cell growth in non-adherent conditions and impairs in vivo metastasis formation. **a**,** b** Relative cell growth in non-adherent conditions after ITGA9 depletion by shRNA. All measurements were taken in triplicates. Values are expressed in percentage and referred to those of the control condition. Statistical significance (*p < 0.05, ***p < 0.001) was obtained after one-way ANOVA test. **c**,** d** Event-free survival (EFS) of mice after intravenous injection of RD and BE(2)-C cells bearing the ITGA9 knockdown, respectively. Statistical significance was obtained after Log-rank survival test. **e**,** f** Qualitative analysis summarizing the number of metastases developed by mice of each group. Relative percentage are indicated within each box. Statistical significance (*p < 0.05) was obtained after Chi-square for trend test

In order to study the effects of ITGA9 depletion, the most sensitive cell lines in terms of invasiveness, RD and BE(2)-C, were selected to perform non-adherent cell growth assays and subsequent in vivo studies. Cells overexpressing sh#1 and sh#2 seeded in non-adherent conditions showed a reduction in cell proliferation after 48 h (Fig. 2a and b). Thus, both cell lines presented reductions in their growth over 30%. The fact that cells lacking ITGA9 were less able to maintain their proliferation in suspension indicated an impairment of their resistance to attachment loss. Moreover, shRNA-mediated ITGA9 downregulation was also able to impair the capability of cells to engraft and develop metastases in mice after tail-vein injection of cancer cells. Thus, the differences in event-free survival were significant for the two models selected (cell line RD for RMS, and BE(2)-C for NB). For the RMS model (Fig. 2c), a clear delay in the detection of metastases and a 30% improvement of event-free survival was observed by the end of the experiment (80% mice of the control group developed metastasis, while only 50% of the shITGA9 group). Likewise, for the NB model (Fig. 2d), a reduction of 30% of metastasis formation was observed (100% mice of the control group developed metastasis, while 70% of the shITGA9 group) at the end point. In this case, all control mice developed metastasis before day 50, while survivors in the group lacking ITGA9 did not show signs of metastases after allowing them to evolve up to 100 days. Similarly, in both models the number of metastasis per animal was reduced in conditions with ITAG9 depletion (Fig. 2e and f).

### Expression analysis of integrin alpha chains in RMS and NB. ITGA9 levels influence patient survival in particular cancer subtypes

**Fig. 3:**
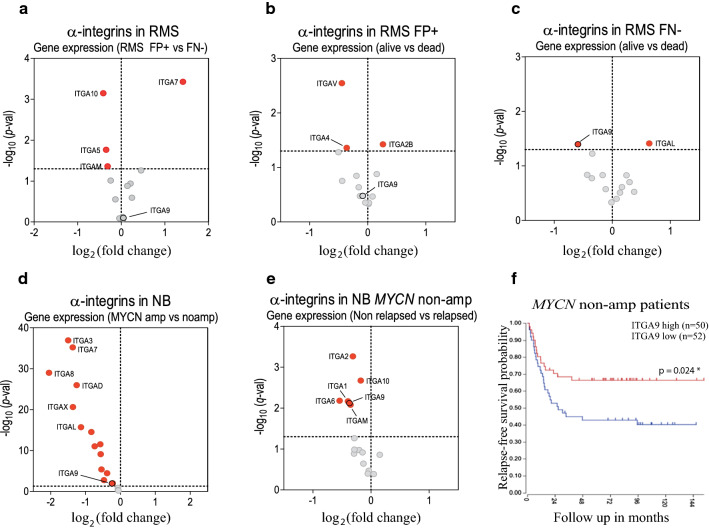
A higher *ITGA9* expression is associated with worse survival in RMS and relapse-free survival in NB. **a** ITGAs expression in a set of RMS patients divided by their molecular subtype (fusion positive (FP +) vs fusion-negative (FN-)). **b**,** c** ITGAs expression in FP + and FN- RMS patients according to their state (alive or dead). **d** ITGAs expression in a set of NB patients depending on their *MYCN* status. **e** ITGAs expression in a non MYCN-amplified set of metastatic patients and **f** their corresponding relapse-free survival curves. (Statistical significance *p* < 0.05 was obtained after Log-rank test.) Tumours whose ITGA9 expression were above the median were classified as high ITGA9, while those below the median were classified as low ITGA9. All data were obtained from public data sets from the R2: Genomics Analysis and Visualization Platform. All Scatter dot plots represent the gene expression for each sample

The analysis of expression data sets revealed a significant correlation between ITGA9 and patient survival, which suggested a potential contribution of this protein to cancer progression in some specific cancer subtypes. Hence, the analysis of the RMS data set of Davicioni et al. 2006 [29] showed no significant differences in terms of ITGA9 expression between fusion positive (FP +) and fusion negative (FN−) RMS tumours (Fig. 3a). In this analysis, it is of note the case of ITGA7, which showed strong overexpression in FP + tumours. ITGA9 showed also no significant differences in the FP + subset when patients were divided into their outcomes of alive or dead (Fig. 3b). In this analysis, 2 integrins (ITGAV and ITGA4) showed significantly lower levels in alive versus dead patients, while ITGA2B showed a significant expression decrease in dead patients. Interestingly, the analysis of the FN-subgroup revealed a notable difference in the expression of ITGA9 between alive patients and those who were not cured (Fig. 3c), being this integrin alpha chain with lower levels in alive patients compared to dead patients, thereby pointing to a preponderant role of this integrin in the progression of cancer within this particular subset of patients (for more complete information see Supplementary Figs. 2, 3 and 4).

The NB data set of Su et al. 2014 [30] revealed a clear trend of integrin alpha chain overexpression in MYCN amplified tumours with significant overexpression of 14 alpha chains in MYCN-amplified tumours. The ITGA9 showed a significant but extremely moderate difference between *MYCN*-amplified and non-*MYCN*-amplified tumours (Fig. 3d). Nonetheless, in the subgroup of patients without *MYCN*-amplification (using Asgharzadeh et al. 2006 [31] data set, specific for metastatic non-*MYCN*-amplified cases) a higher ITGA9 expression was observed in relapsed patients when compared with the subgroup without relapse (Fig. 3e). Beyond ITGA9, also other integrins showed higher levels in relapsed patients (ITGA2, 10, 1, 6 and M). Moreover, the analysis of relapse-free survival (available for this subset) in MYCN non-amplified patients (comparing ITGA9 high- and low-level groups) revealed a significant decrease of relapse-free survival from 0.68 to 0.40 after 144 months of follow up (Fig. 3f). For more complete information see Supplementary Figs. 5, 6 and 7.

### Design and synthesis of a peptide able to interfere with the activity of ITGA9

**Fig. 4:**
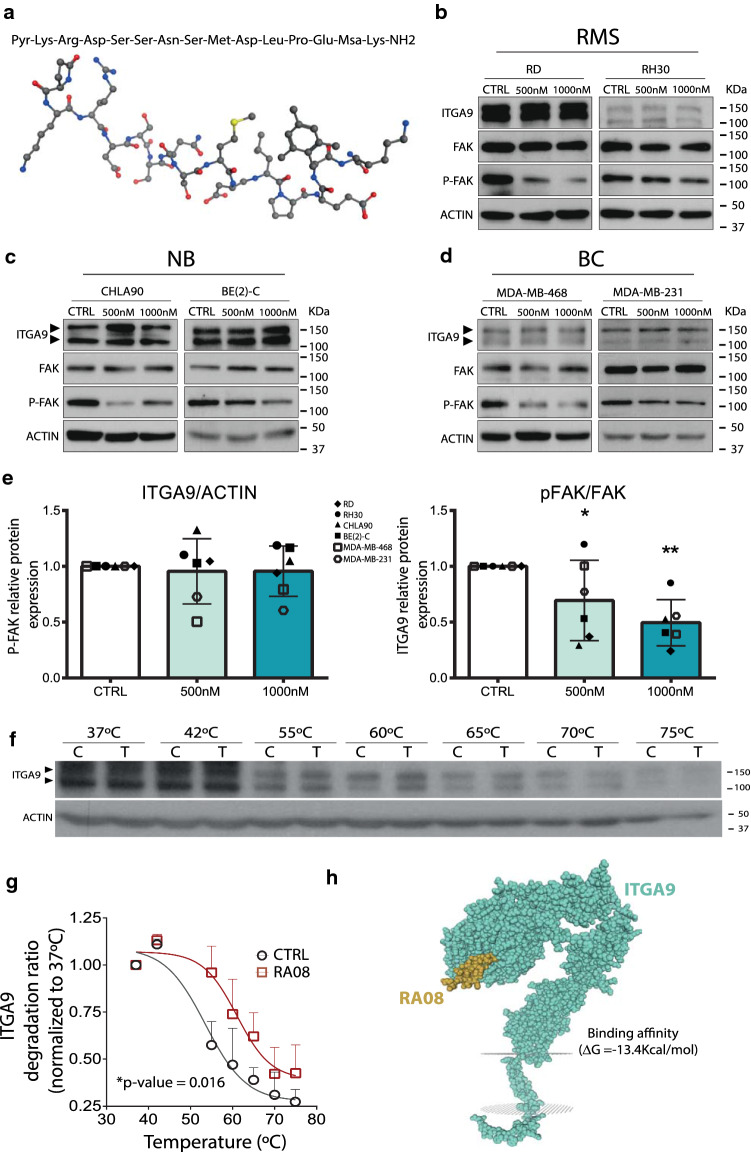
Treatment with RA08 does not affect ITGA9 or total FAK expression but decreases FAK phosphorylation. **a** Schematic representation of the structure of the synthetic peptide RA08. **b**–**d** Representative immunoblots showing ITGA9, total-FAK and phosphorylated-FAK expression levels in RMS (**b**), NB (**c**) and BC (**d**) cell lines, after 48 h of RA08 treatment at two different doses (500 nM and 1000 nM). **e** Densitometric analysis of western blots (shown in **b**–**d**) showing ITGA9 levels and the ratio P-FAK/FAK. Mean values were derived from single experiments performed in 6 different cell lines**.** Statistical significance (*p < 0.05, **p < 0.01, ***p < 0.001) was obtained after one-way ANOVA test. **f** Thermal shift assay, on which cells were exposed to a temperature gradient (from 37 to 75 °C) and protein extracts were resolved into a Western blot to measure changes of ITGA9 degradation in presence (T) or absence (C) of RA08. **g** Plot showing the differences observed in ITGA9 degradation in the thermal shift assay. Data were derived from 3 independent Western blots, with significant differences observed (*p* = 0.016, paired Student *t* test). **h** Representative 3D model of the interaction between RA08 and ITGA9 generated by LZerD, PRODIGY and SWISS-MODEL

In this context, as the disintegrin domain of ADAMs plays a crucial role in interacting with integrins, RA08 was designed as a short peptide aimed at inhibiting the interaction between ITGA9 and ADAM proteins. Thus, RA08 is a 15 amino acid peptide (Pyr-Lys-Arg-Asp-Ser-Ser-Asn-Ser-Met-Asp-Leu-Pro-Glu-Msa-Lys-NH2) derived from the disintegrin domain of the native sequence of ADAM-12 (-Arg-Asp-Ser-Ser-Asn-Ser-Cys-Asp-Leu-Pro-Glu-Phe-). RA08 has been modified at the N-terminus with the inclusion of an unnatural amino acid (pyroglutamic acid, Pyr), together with other modifications such as the substitution of Cys by Met, the substitution of Phe by 3-mesitylalanine (2,4,6-trimethylphenylalanine, Msa), and the incorporation of two Lys at the N- and C-termini of the sequence. All these modifications aim to increase its stability in plasma and improve its pharmacological properties for inhibiting the ITGA9-ADAM interaction, with the ultimate aim of impairing cancer cell invasion and metastasis. Additionally, the incorporation of two further Lys residues, one at the N-terminus and another at the C-terminus, was performed to improve the peptide sequence’s solubility when formulated in physiological saline (Fig. 4a).

### Treatment with RA08 produced a decrease in FAK-phosphorylation in vitro

In concordance with the results obtained with shRNA against ITGA9, treatment with RA08 produced a reduction in FAK phosphorylation (at Tyr397) in RMS and NB cell lines. In this case, since it has been previously suggested that ITGA9 played a role in breast cancer (BC) progression, BC cell lines were also included to test the possibility of the compound in this neoplasia. The results showed a remarkable reduction in FAK phosphorylation in RA08-treated cells in a dose-dependent manner, whereas total FAK and ITGA9 levels were unaltered (Fig. 4b to e).

### Analysis of the RA08/ITGA9 binding

Experimental data using CETSA protocol revealed a clear difference in the ITGA9 degradation in the presence or absence of RA08. The results showed a statistically significant differential degradation rate (*p* value: 0.016), observable from 55ºC onward, thereby suggesting that RA08 binds ITGA9 and protects it against thermic degradation (Fig. 4f and g).

Additionally, the in silico analysis revealed also a high probability of binding between RA08 and ITGA9 (on its beta-propeller domain) with a binding affinity prediction value of − 13.4 Kcal mol^−1^ (Fig. 4h). Interestingly, the same analysis performed between ADAM12 and ITGA9 rendered similar results with a binding affinity prediction value of − 13.1 kcal mol^−1^ (Supplementary Fig. 8) between beta-propeller domain for ITGA9 and the domain of ADAM12 that contains the peptide that inspired RA08. For more information and interactive format of the 3D structure pbd files, see Supplementary File 1 (RA08/ITGA9) and 2 (ADAM12/ITGA9).

### Treatment with RA08 significantly reduced cell invasiveness

**Fig. 5:**
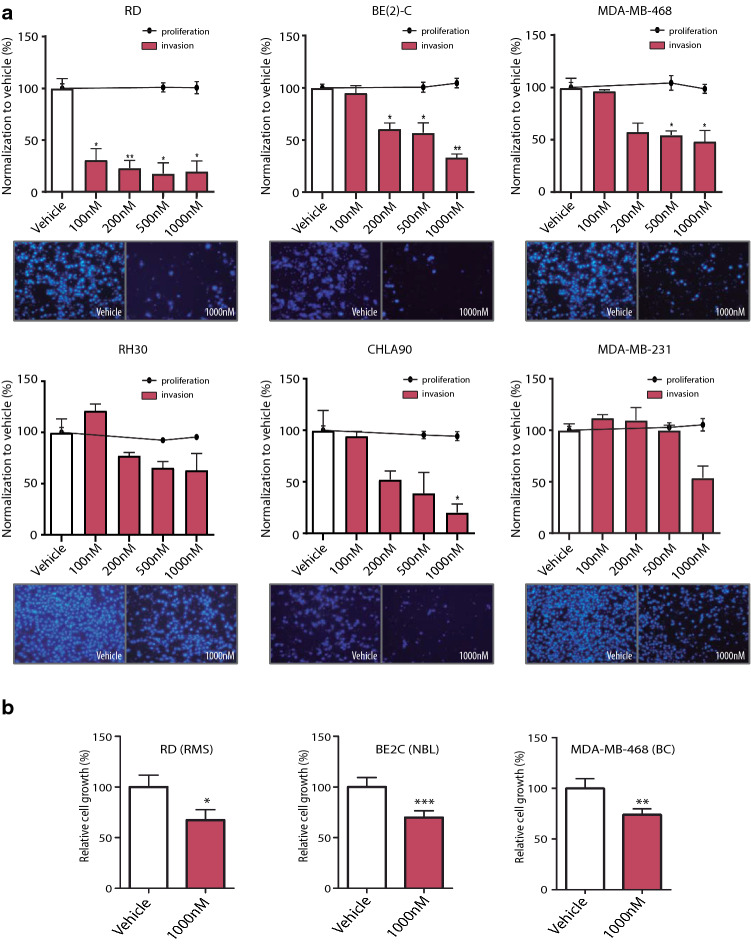
Treatment with RA08 significantly reduces cell invasiveness. **a** Relative cell invasiveness and cell proliferation after treatment with increasing concentrations of RA08 (100 nM, 200 nM, 500 nM and 1000 nM) in RMS (RD and RH30), NB (BE(2)-C and CHLA90) and BC (MDA-MB-468 and MDA-MB-231). Bars represent cell invasion, whereas cell proliferation is represented by dots and lines. Representative images of the Transwell-invasion assays, including control cells and cells treated with 1000 nM RA08 are also shown below each plot. **b** Relative cell growth in non-adherent conditions after 48 h of RA08 treatment at 1000 nM. **a**,** b** All measurements were taken in triplicates. All values are expressed in percentage and referred to those of the control condition. Statistical significance (*p < 0.05, **p < 0.01, ***p < 0.001) was obtained after one-way ANOVA test

Treatment with RA08 produced a significant reduction in cell invasiveness in the majority of RMS, NB and BC cell lines, with RA08 concentrations ranging from 100 to 1000 nM. The effects of RA08 were tested in two cell lines for each of the cancers included in this work (Fig. 5a). In RMS, the RD cell line was very sensitive, with an invasiveness reduction over 50% even at the lowest dose (100 nM). Conversely, in the RH30 cell line, which showed very low ITGA9 expression (Supplementary Fig. 1a), the response to the drug was moderate. The NB cell lines also showed marked sensitivity to the drug, with significant reductions in cell invasiveness for both cell lines (over 50% at the higher doses). Finally, the BC cell line MDA-MB-468 showed a clear reduction in invasiveness under treatment (around 50% reduction), while the MDA-MB-231 cell line showed only a moderate reduction at the highest treatment dose. On the other hand, the treatment with RA08 did not produce effects on cell growth, thereby suggesting no toxic effect of the compound. Conversely, incubation with an RGD peptide (the most representative binding motif involved in the interactions of ECM proteins with integrins) promoted a significant decrease of cell growth (Supplementary Fig. 9) as well as a variable but significant reduction in cell invasiveness.

Similarly, treatment with RA08 reduced cell growth when cells were cultured in anchorage-independent conditions, concurring with the previous results obtained with the genetic depletion of ITGA9. Thus, in the 3 cell lines analysed, a reduction of ~ 30% in anchorage-independent cell growth was observed in the presence of RA08 at 1000 nM (Fig. 5b).

### Treatment with RA08 reduced in vivo experimental metastasis

**Fig. 6:**
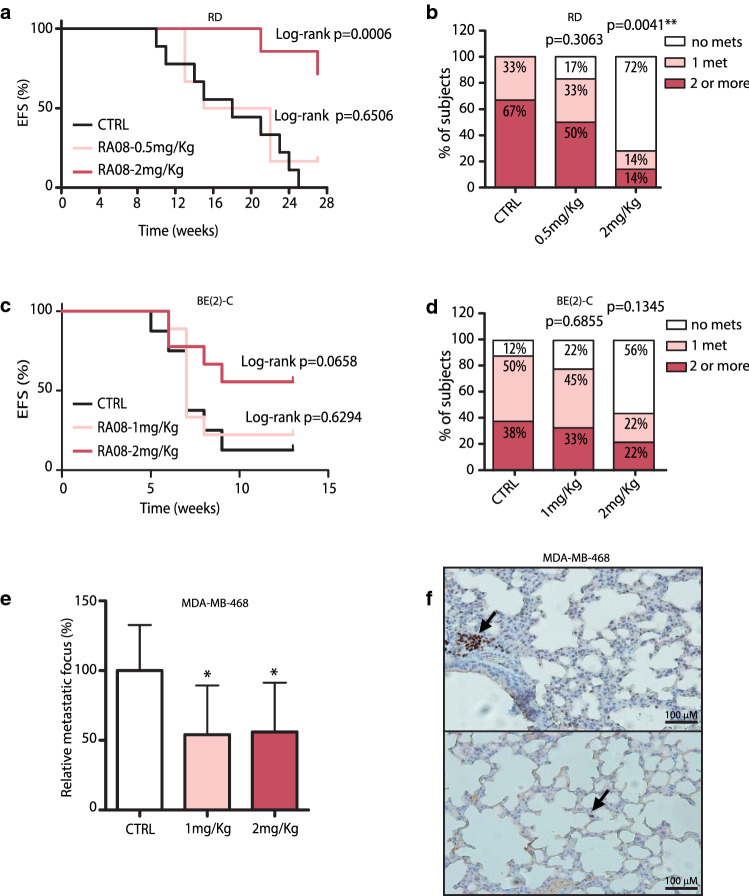
Treatment with RA08 impairs in vivo experimental metastasis formation. **a** Event-free survival (EFS) of mice after intravenous injection of RD cells and treatment with two doses of RA08 (0.5 mg/kg and 2 mg/kg) three times per week. **c** Event-free survival (EFS) of mice after intravenous injection of BE(2)-C cells and treatment with two doses of RA08 (1 mg/kg and 2 mg/kg) three times per week. In (**a**) and (**c**) statistical significance was obtained after Log-rank survival test. **b**,** d** Qualitative analysis summarizing the number of metastases developed by mice of each group. Relative percentage are indicated within each box. Statistical significance (**p < 0.01) was obtained after Chi-square for trend test. **e** Quantification of metastatic foci in the lungs of mice after intravenous injection of MDA-MB-468 cells and treatment with two doses of RA08 (1 mg/kg and 2 mg/kg) three times per week. Quantification was based on GATA3 expression. Values are expressed as percentage and referred to those of the control condition. Statistical significance (*p < 0.05) was obtained after one-way ANOVA. **f** Representative images of GATA3 immunohistochemical staining in lung sections from mice treated with the vehicle (upper) and the higher RA08 dose (lower). Arrows indicate GATA3 + cells which were considered metastatic cells. Bar: 100 µm

With the aim of evaluating the therapeutic potential of RA08 in vivo murine metastatic experimental models were established by tail vein injection. In the RMS model (Fig. 6a), all control mice developed metastases by week 25 post-injection. The first metastasis appeared at week 10 in this group. The lower RA08 dose (0.5 mg/Kg) was scarcely effective, and the majority of RA08-treated mice also developed metastases (83%). Conversely, mice treated with RA08 at 2 mg/Kg showed a clear reduction in metastases formation, with a clear delay in the appearance of the first metastasis (reported at week 21), and only 28% of cases metastasizing. The number of metastatic foci detected at necropsy was also reduced by RA08. While most mice developed 2 or more metastases in the control group, only 1 mouse developed 2 or more metastases in the higher dose group (Fig. 6b).

Likewise, the NB model (Fig. 6c) revealed a similar trend, with effectivity in terms of protection against metastasis of the higher dose (2 mg/Kg), and no differences for the lower dose (in this case, 1 mg/Kg). The 2 mg/Kg dose provided protection against metastasis in 56% of mice and the number of metastatic foci detected was also reduced with the treatment (Fig. 6d).

Finally, the BC model showed a diffuse pattern of invading cells and it was not possible to quantify the number of macroscopic lesions. In this case, with the aim of evaluating metastatic foci in mouse lungs, immunohistochemical staining of GATA3 was used as a marker of tumour cells. Although the quantification procedure was different due to the diffuse nature of the metastatic cells, the results also showed a reduction in metastatic foci to half for the two doses analysed (1 mg/kg and 2 mg/kg) (Fig. 6e and f).

## Discussion

It is well known that the onset of metastasis very often involves crossing a boundary beyond which the probability of cure becomes clearly lower. The fact that most of cancer-related deaths are attributed to metastases, but, unfortunately, treatments for their blockade have not yet been established, is a tremendous paradox. Probably, the more advanced studies in this direction were focused on inhibiting angiogenesis [36, 37], matrix metalloproteinases [38] or impairing tumour cell embolization by reducing clumping with platelets [39]. Unfortunately, none of these have successfully reached the clinical setting as yet; therefore, the development of treatments aimed at blocking metastasis is a pending clinical need that could lead to an improvement in survival.

A noticeable variable of metastasis is its temporal course, which strongly depends on cancer type. For instance, in small cell lung cancer and pancreatic cancer, metastatic disease often appears at the time of (or shortly after) the initial diagnosis, whereas many patients with breast and prostate cancers, among many others, tend to show metastasis after a prolonged period of time [40]. These obvious clinical differences have raised an important question of whether this period of dormancy offers a therapeutic opportunity to control metastatic disease [41]. Moreover, circulating tumour cells in paediatric solid tumours, such as RMS and NB, are quite common in compartments such as peripheral blood or bone marrow and are thought to contain, at least potentially, cells that may originate metastases, often a long time after diagnosis. Despite some controversy on the clinical significance of these cells, there is a high detection rate in patients with paediatric solid tumours associated with a higher probability to develop metastases [42, 43]. Despite little being known about the mechanism by which cancer cells enter and maintain a dormant state, and what triggers the resumption of aggressive growth, the interaction of cells with the extracellular matrix environment (with particular reported roles for Type I collagen, fibronectin and integrins and subsequent activation of FAK [44, 45]) appears to facilitate re-entry to the metastatic phenotype [46, 47]. Although it is possible that other actors may also be able to influence P-FAK levels, the effects exerted on cells by RA08 (or ITGA9 genetic inhibition) led to the impairment of FAK phosphorylation. Thus, we can hypothesize the possibility that, in our RMS, NB and BC animal experimental models, ITGA9 impairment would be able to hinder cell cycle re-entry once cells reached the niche tissue, thereby pointing to an inhibition of cell reactivation at this stage. However, this is not the only point at which RA08 or ITGA9 genetic inhibition may interfere with metastasis, since we have also detected a decrease in non-adherent cell growth after treatment with RA08. Integrin-family proteins have been extensively involved in anoikis resistance [48, 49], enabling cells to endure the lack of attachment before the colonization of new potential niche tissues. Moreover, the fact that ITGA9 inhibition or treatment with RA08 clearly impairs in vitro invasiveness, strongly suggests that the migration step through the extracellular matrix that cells must perform to reach blood vessels and, in turn, to reach the niche tissue after extravasation, could also be strongly compromised. Therefore, we think that the possible effect of ITGA9 inhibition (genetic or pharmacological) has to be viewed as sequential interferences of a multistep process, rather than a complete blockade of a single step.

The experiments herein provided revealed a strong dependency on ITGA9 in several steps of metastasis, even though the experimental tail vein models provided skip initial steps of the process and, therefore, ITGA9 role may be even more extensive. The RGD peptide used as a reference promoted similar effects on cell invasiveness. However, the strong concomitant reduction observed in cell growth pointed to a higher toxicity and to a less-specific activity, probably caused by the wide range of integrins and ligands potentially affected by RGD blocking. This reinforces our approach and suggest a greater specificity of ITGA9 blocking as well as lower toxicity. Interestingly, a strong variability in the response to the inhibition of ITGA9 among different cell lines was detected. Particularly, RD and BE(2)-C cell lines were clearly more sensitive than CHLA90 (slightly sensitive), or RH30 (moderately sensitive). These differences observed among cell lines pointed to a diversity of response probably caused by the molecular background of each cell line, ITGA9 basal expression (Supplementary Fig. 1a and b) and the histology of the tumours from which were derived. Interestingly, the patient’s gene expression data presented (Fig. 3) also pointed to a great disparity in the correlation between ITGA9 levels and survival. Thus, only in some subtypes, levels of ITGA9 correlate with mortality. Intriguingly, the outcomes of patients belonging to the most aggressive subtypes (such as fusion-positive in RMS and *MYCN*-amplified in NB) are completely independent of ITGA9 levels. Conversely, in the case of patients classified as fusion-negative in RMS and non-*MYCN* amplified in NB, the outcomes showed dependency on ITGA9 levels. Consequently, a blocking of its function may hinder the development of metastasis in these specific subtypes. Interestingly, fusion-negative RMS and non-*MYCN*-amplified NB are considered to have better prognosis compared with less favourable subgroups of patients; however, unresolved questions persist regarding the optimal management for these groups of patients given that some cases can still relapse and/or metastasize. The fact that, in RMS and NB, ITGA9 can discriminate a subgroup of patients with worse prognosis should be considered for subsequent studies, as this new set of patients with worst prognosis would be candidates for putative ITGA9-based anti-metastatic therapy or, at least, for an intensification of treatment owing to their putative propensity to metastasize. Two BC cell lines were included in this part of the study to test the possible applicability of the compound to other neoplasia. In these cell lines, variability was also observed between the 2 cell lines analysed, with the cell line MDA-MB-468 showing significant sensitivity to the compound whilst in the other (MDA-MB-231), effects were only observed at the highest dose (1000 nM). Both cell lines correspond to triple negative BC, but relative ITGA9 expression is notably higher in MDA-MB-468 (Supplementary Fig. 1c), thereby suggesting that the expression level may correlate with sensitivity to treatment with RA08.

The RA08 compound, designed to inhibit ITGA9, produced a very clear reduction of FAK phosphorylation, suggesting a specificity for integrins, since FAK is one of the main downstream effectors of this family of proteins. However, we cannot exclude activity of the RA08 compound on other members of the family, particularly ITGA4, which shares high homology with ITGA9. The fact that compound RA08 showed no effect on cell proliferation is noteworthy since it may indicate a very low or inexistent toxicity at the doses tested. Likewise, no signs of toxicity were observed in murine models at the concentrations studied (up to 2 mg/kg), although clear effects upon reduction of metastasis were found in RMS, NB and BC in vivo models. Beyond the effects of RA08 on metastasis, the low toxicity of the compound makes it particularly interesting, since a putative therapy for metastasis prevention is expected to be long-term, probably spanning several months and, therefore, its possible future implantation is going to be enormously facilitated if the compounds used show a complete absence of associated non-desirable side effects.

Taken together, our results indicate an important role of ITGA9 in the establishment of metastasis and present RA08 as a new compound able to prevent its origin in RMS and NB models, with potential applicability in a wide range of tumour types. In addition, the prognostic value of ITGA9 expression is also depicted, emphasizing the potential of this protein as prognostic factor in some subsets of patients.

## Supplementary Information

Below is the link to the electronic supplementary material.

ITGA9 is expressed in RMS, NB and BC cell lines. Representative immunoblot showing ITGA9 protein levels in a RMS, b NB and c BC cell lines (PDF 845 KB)

Comparative gene expression analysis of alpha integrins in RMS by its molecular subtype (fusion-positive (FP+) vs fusion-negative (FN-)). Scatter dot plots representing the gene expression for each sample. Lines and error bars represent the mean ± CI 95%. No data was available for ITGA3, ITGA11, ITGAD and ITGAX. Statistical significance (*p < 0.05, ***p < 0.001) was obtained after two-tailed t-test (PDF 71 KB)

Comparative gene expression analysis of alpha integrins in RMS FP+ by its state (alive or dead). Scatter dot plots representing the gene expression for each sample. Lines and error bars represent the mean ± CI 95%. No data was available for ITGA3, ITGA11, ITGAD and ITGAX. Statistical significance (*p < 0.05, **p < 0.01) was obtained after one-tailed t-test (PDF 46 KB)

Comparative gene expression analysis of alpha integrins in RMS FN- by its state (alive or dead). Scatter dot plots representing the gene expression for each sample. Lines and error bars represent the mean ± CI 95%. No data was available for ITGA3, ITGA11, ITGAD and ITGAX. Statistical significance (*p < 0.05) was obtained after one tailed t-test (PDF 54 KB)

Gene expression analysis of alpha integrins in NB MYCN amplified compared to MYCN non-amplified. Scatter dot plots representing the gene expression for each sample. Lines and error bars represent the mean ± CI 95%. Statistical significance (*p < 0.05, **p < 0.01, ***p < 0.001) was obtained after two-tailed t-test (PDF 155 KB)

Gene expression analysis of alpha integrins in relapsed NB MYCN non-amplified compared to non-relapsed. Scatter dot plots representing the gene expression for each sample. Lines and error bars represent the mean ± CI 95%. No data was available for ITGA11 and ITGAD. Statistical significance (*p < 0.05) was obtained after one-tailed t-test (PDF 57 KB)

Relapse-free survival curves of NB MYCN non-amplified patients considering each alpha integrin expression. For each alpha integrin analysed, samples (n = 102) were separated in high or low expression considering the median gene expression. Statistical significance was obtained after Log-rank test to compare survival distributions (PDF 158 KB)

Supplementary file8 (PDF 110 KB)

Relapse-free survival curves of NB MYCN non-amplified patients considering each alpha integrin expression. For each alpha integrin analysed, samples (n = 102) were separated in high or low expression (PDF 11810 KB)

Treatment with RGD peptide significantly reduces cell invasiveness and cell growth. a. Relative cell invasiveness and cell proliferation after treatment with increasing concentrations of RGD peptide (100nM, 200nM, 500nM and 1000nM) in RMS (RD and RH30), NB (BE(2)-C and CHLA90) and BC (MDA-MB-468 and MDA-MB-231). Bars represent cell invasion (significance indicated with asterisks), whereas cell proliferation is represented by dots and lines (significance indicated with hashes). Representative images of the Transwell-invasion assays, including control cells and cells treated with 1000nM are also shown below each plot. All measurements were taken in triplicates. All values are expressed in percentages and referred to those of the control condition. Statistical significance (* or # p<0.05, ** or ## p<0.01, *** or ### p<0.001) was obtained after one-way ANOVA test. b. Molecular structure, name and chemical formula of the RGD peptide used for this experiment (PDF 5713 KB)

File containing the 3D structure of RA08 and ITGA9 predicted interaction from LZerD protein docking suite platform (PBD 655 KB)

File containing the 3D structure of ADAM12 and ITGA9 predicted interaction from LZerD protein docking suite platform (PBD 1174 KB)

## Data Availability

This study does not include data deposited in external repositories.
