# Supplementary figures and images for: Integrin alpha9 emerges as a key therapeutic target to reduce metastasis in rhabdomyosarcoma and neuroblastoma
Source: Cell Mol Life Sci. 2022 Oct 11;79(11):546. doi: 10.1007/s00018-022-04557-y (PMC9553833; doi:10.1007/s00018-022-04557-y)

a

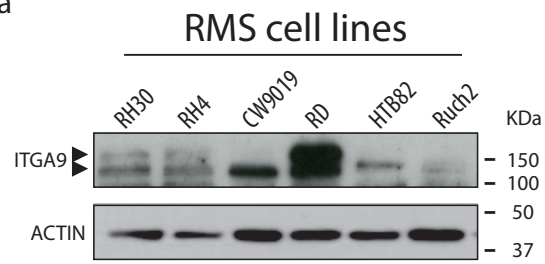

b

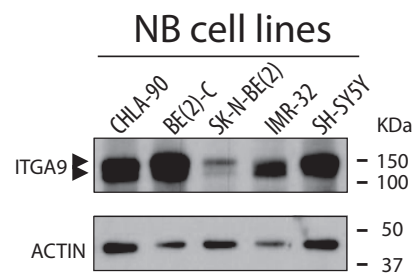

c

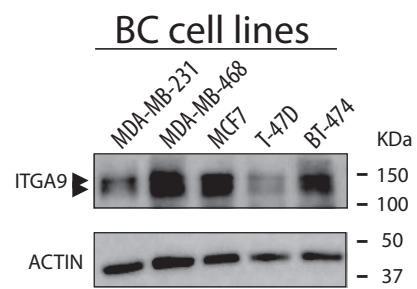

Supplement: Supplementary file 1 — ITGA9 is expressed in RMS, NB and BC cell lines. Representative immunoblot showing ITGA9 protein levels in a RMS, b NB and c BC cell lines (PDF 845 KB) [file 18_2022_4557_MOESM1_ESM.pdf]

# Supplementary Figure 2

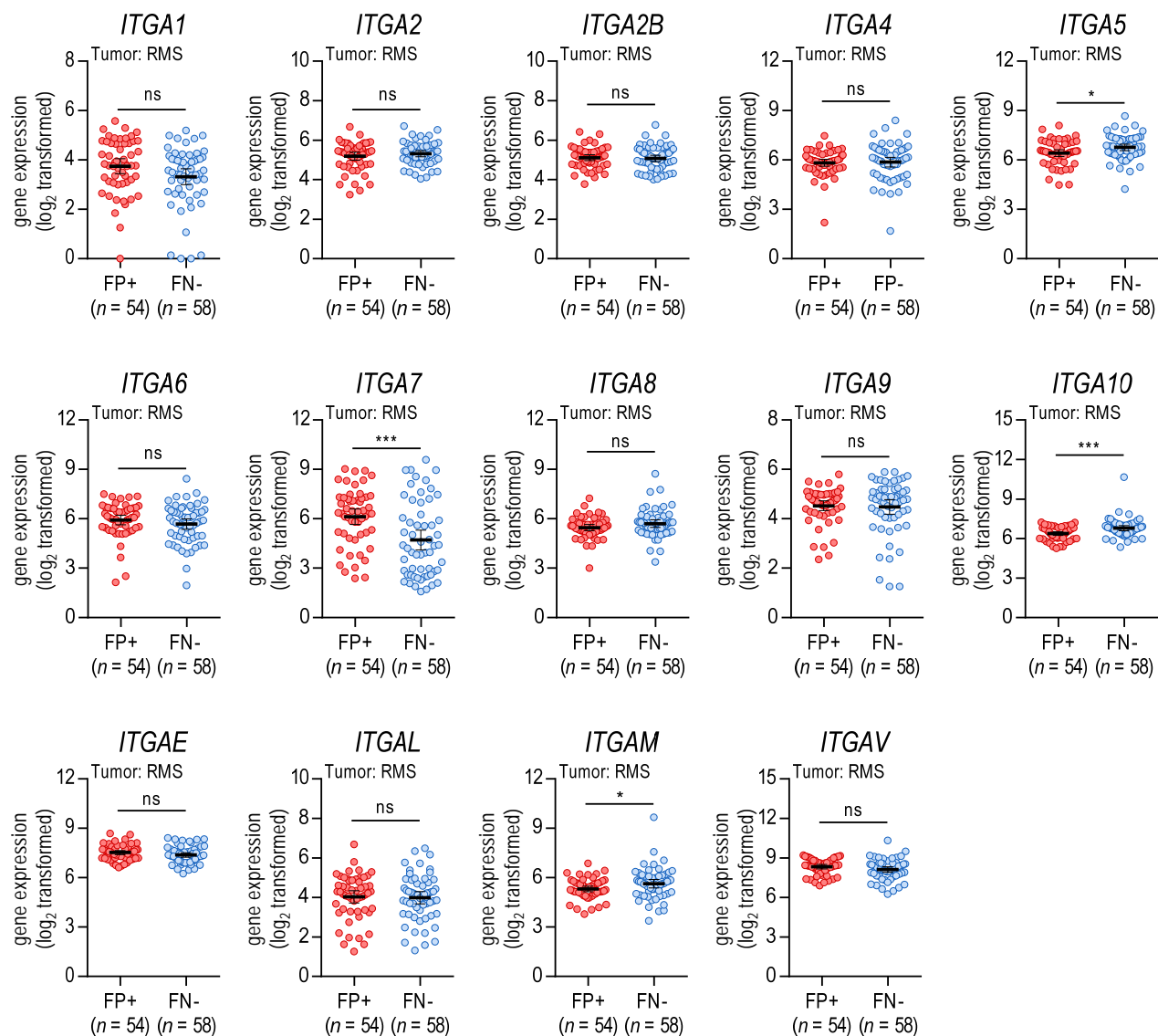

Supplement: Supplementary file 2 — Comparative gene expression analysis of alpha integrins in RMS by its molecular subtype (fusion-positive (FP+) vs fusion-negative (FN-)). Scatter dot plots representing the gene expression for each sample. Lines and error bars represent the mean ± CI 95%. No data was available for ITGA3, ITGA11, ITGAD and ITGAX. Statistical significance (*p < 0.05, ***p < 0.001) was obtained after two-tailed t-test (PDF 71 KB) [file 18_2022_4557_MOESM2_ESM.pdf]

# Supplementary Figure 3

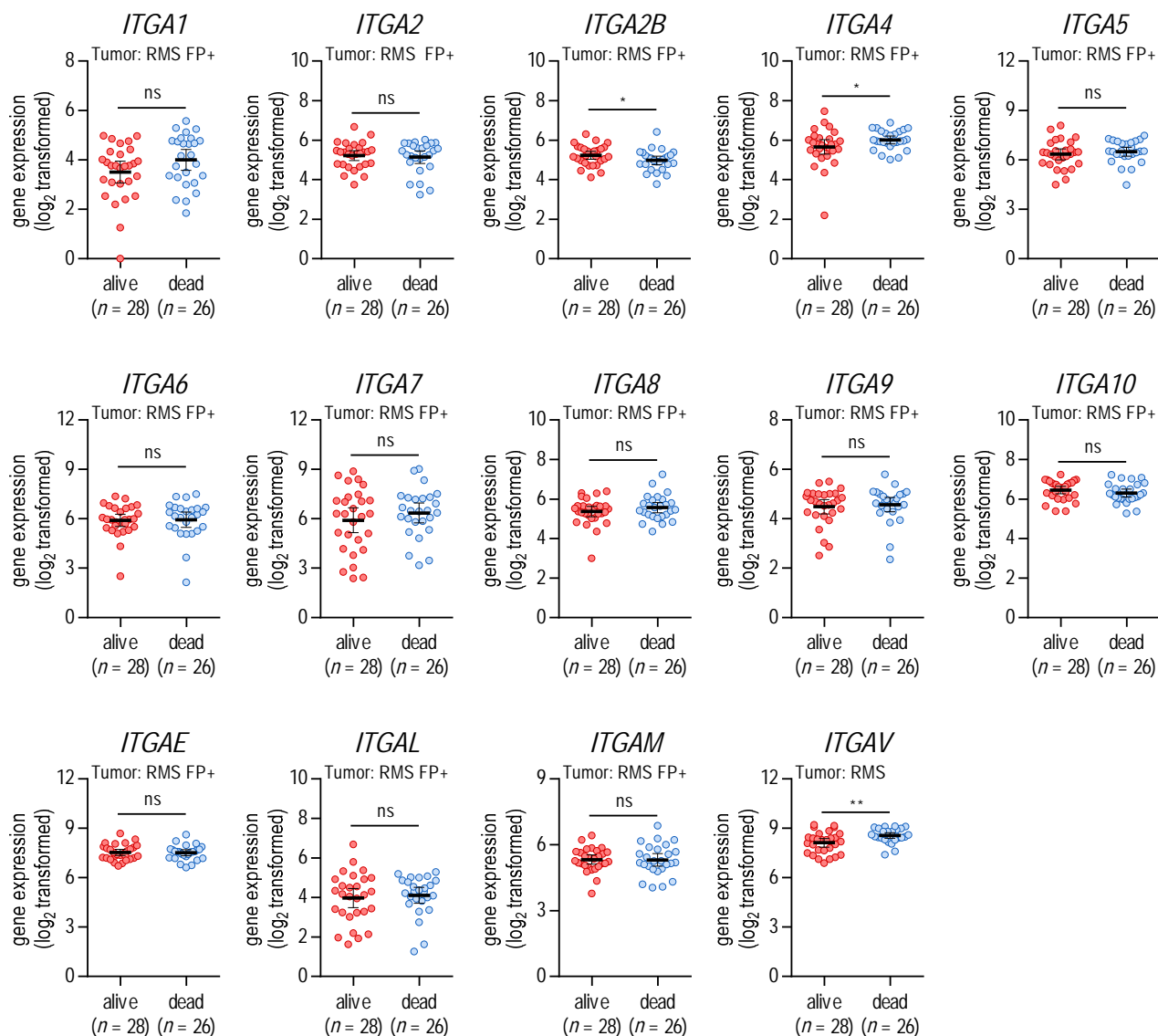

Supplement: Supplementary file 3 — Comparative gene expression analysis of alpha integrins in RMS FP+ by its state (alive or dead). Scatter dot plots representing the gene expression for each sample. Lines and error bars represent the mean ± CI 95%. No data was available for ITGA3, ITGA11, ITGAD and ITGAX. Statistical significance (*p < 0.05, **p < 0.01) was obtained after one-tailed t-test (PDF 46 KB) [file 18_2022_4557_MOESM3_ESM.pdf]

## Supplementary Figure 4

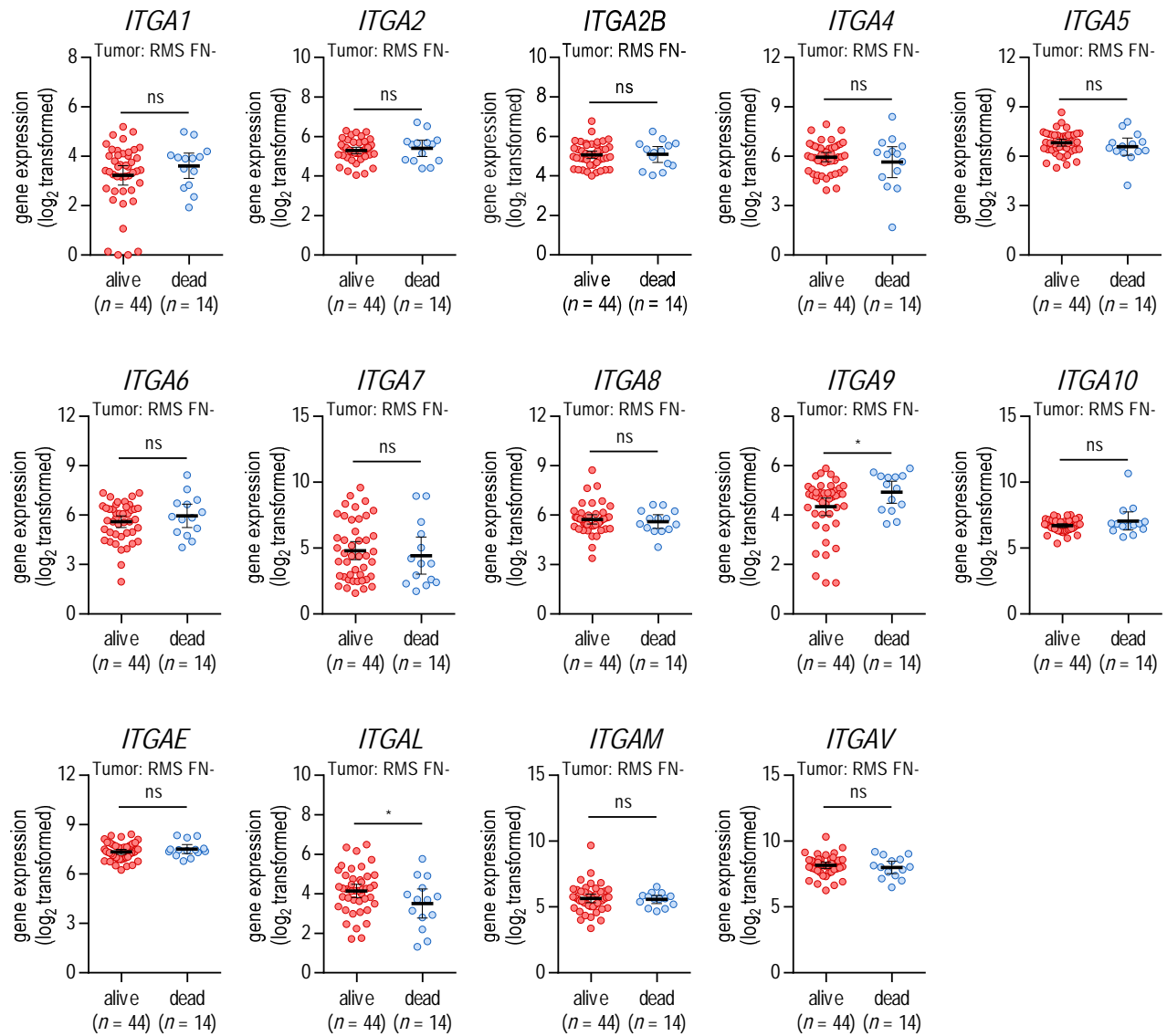

Supplement: Supplementary file 4 — Comparative gene expression analysis of alpha integrins in RMS FN- by its state (alive or dead). Scatter dot plots representing the gene expression for each sample. Lines and error bars represent the mean ± CI 95%. No data was available for ITGA3, ITGA11, ITGAD and ITGAX. Statistical significance (*p < 0.05) was obtained after one tailed t-test (PDF 54 KB) [file 18_2022_4557_MOESM4_ESM.pdf]

# Supplementary Figure 5

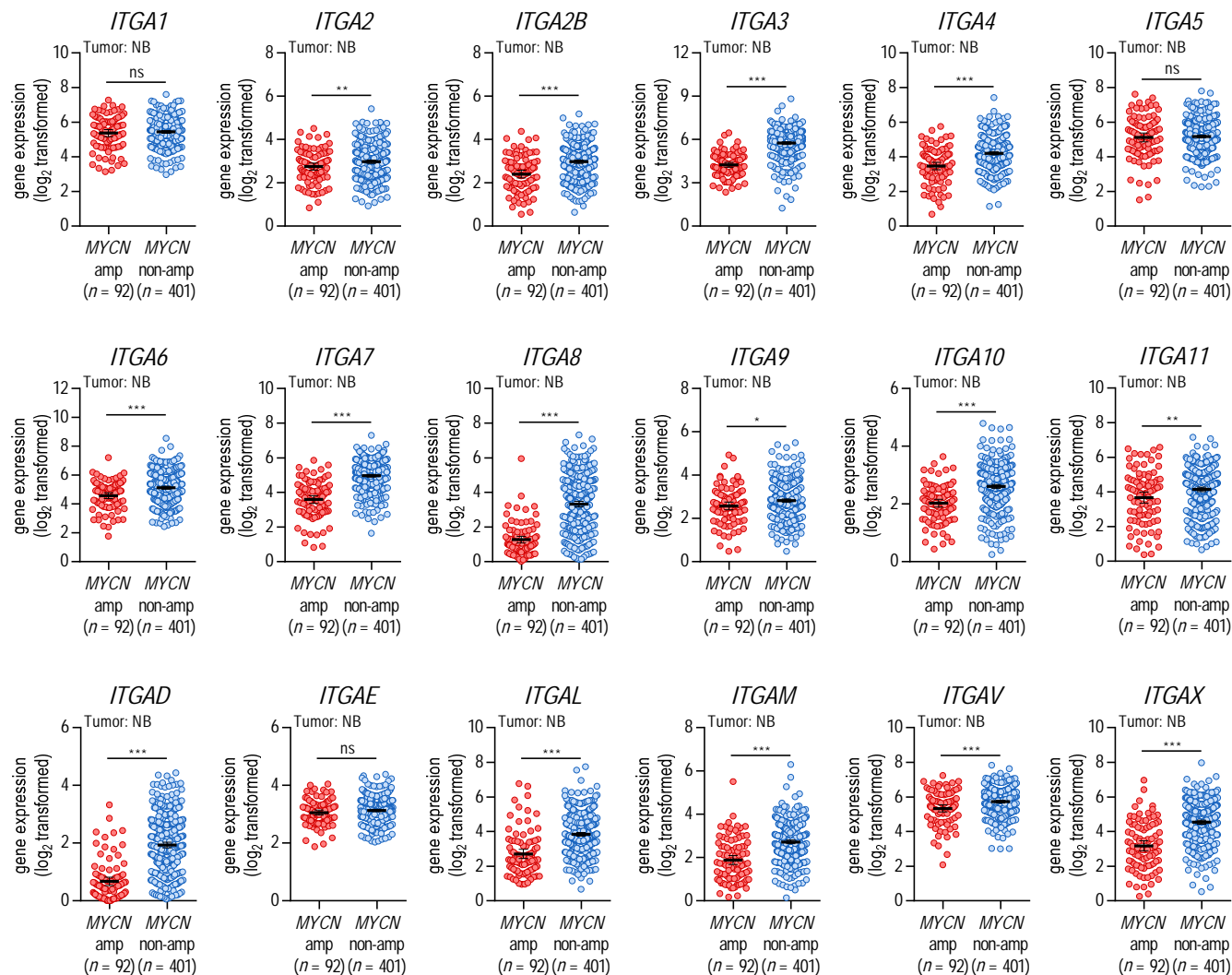

Supplement: Supplementary file 5 — Gene expression analysis of alpha integrins in NB MYCN amplified compared to MYCN non-amplified. Scatter dot plots representing the gene expression for each sample. Lines and error bars represent the mean ± CI 95%. Statistical significance (*p < 0.05, **p < 0.01, ***p < 0.001) was obtained after two-tailed t-test (PDF 155 KB) [file 18_2022_4557_MOESM5_ESM.pdf]

## Supplementary Figure 6

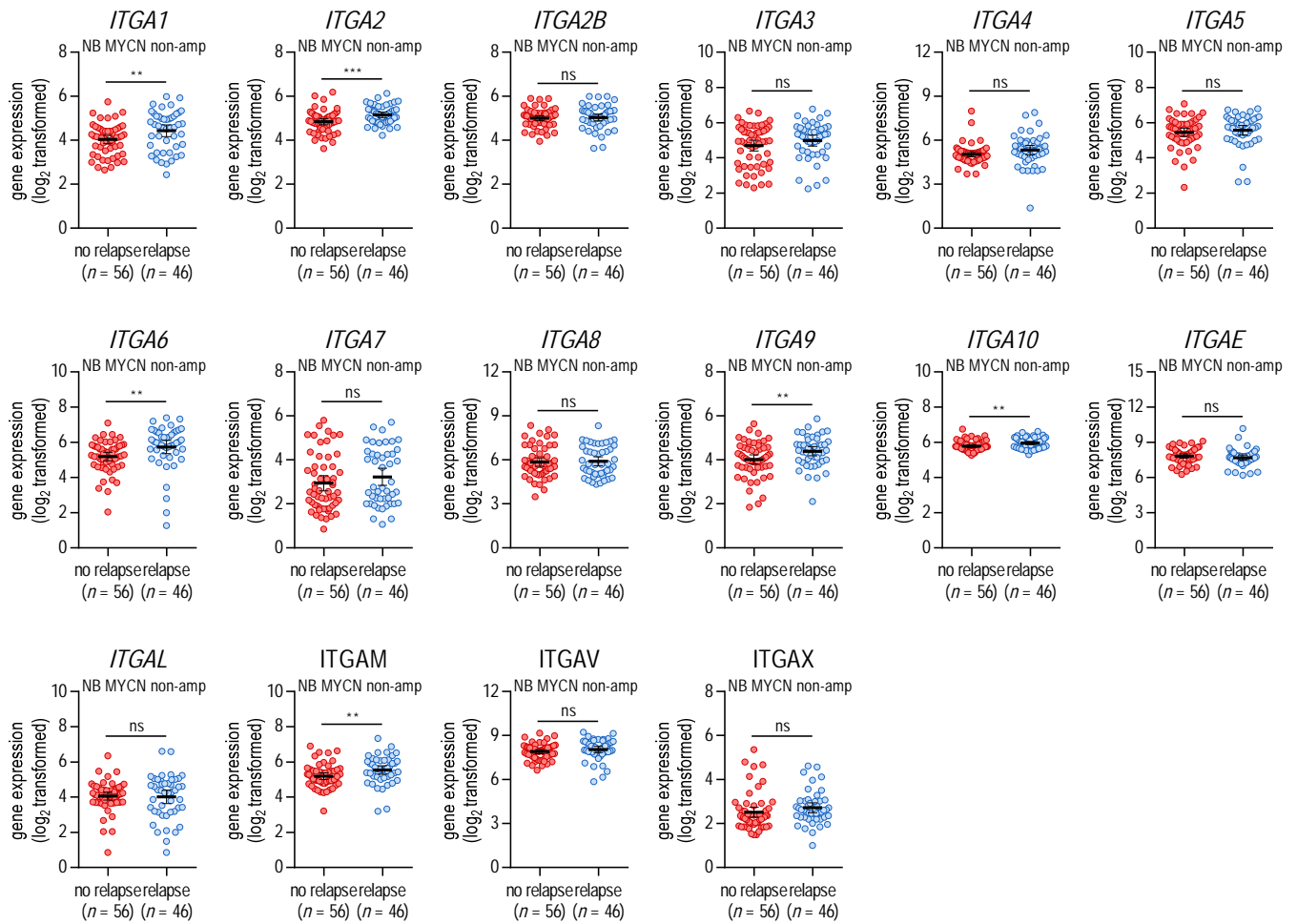

Supplement: Supplementary file 6 — Gene expression analysis of alpha integrins in relapsed NB MYCN non-amplified compared to non-relapsed. Scatter dot plots representing the gene expression for each sample. Lines and error bars represent the mean ± CI 95%. No data was available for ITGA11 and ITGAD. Statistical significance (*p < 0.05) was obtained after one-tailed t-test (PDF 57 KB) [file 18_2022_4557_MOESM6_ESM.pdf]

## Supplementary Figure 7 (part I)

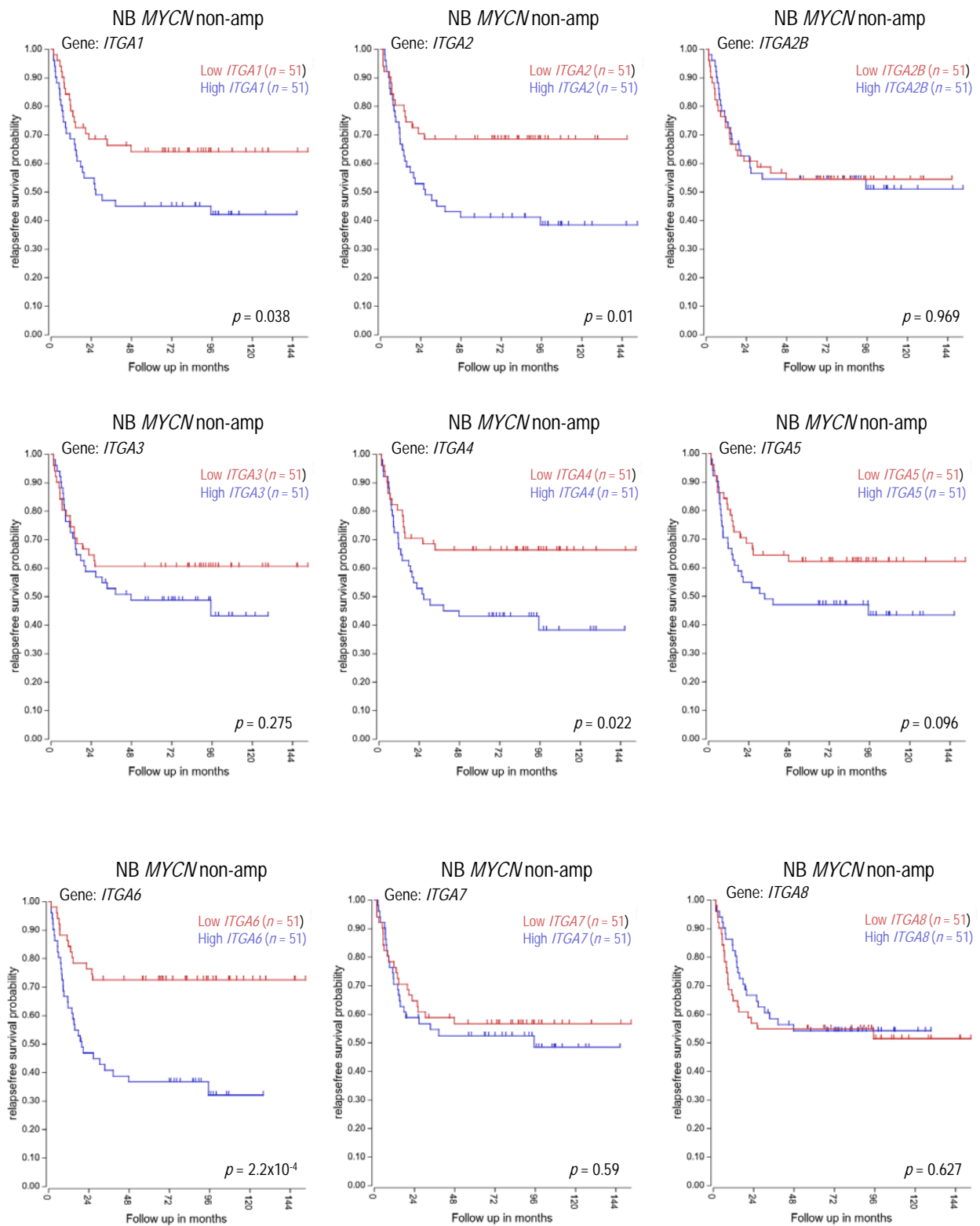

Supplement: Supplementary file 7 — Relapse-free survival curves of NB MYCN non-amplified patients considering each alpha integrin expression. For each alpha integrin analysed, samples (n = 102) were separated in high or low expression considering the median gene expression. Statistical significance was obtained after Log-rank test to compare survival distributions (PDF 158 KB) [file 18_2022_4557_MOESM7_ESM.pdf]

## Supplementary Figure 7 (part II)

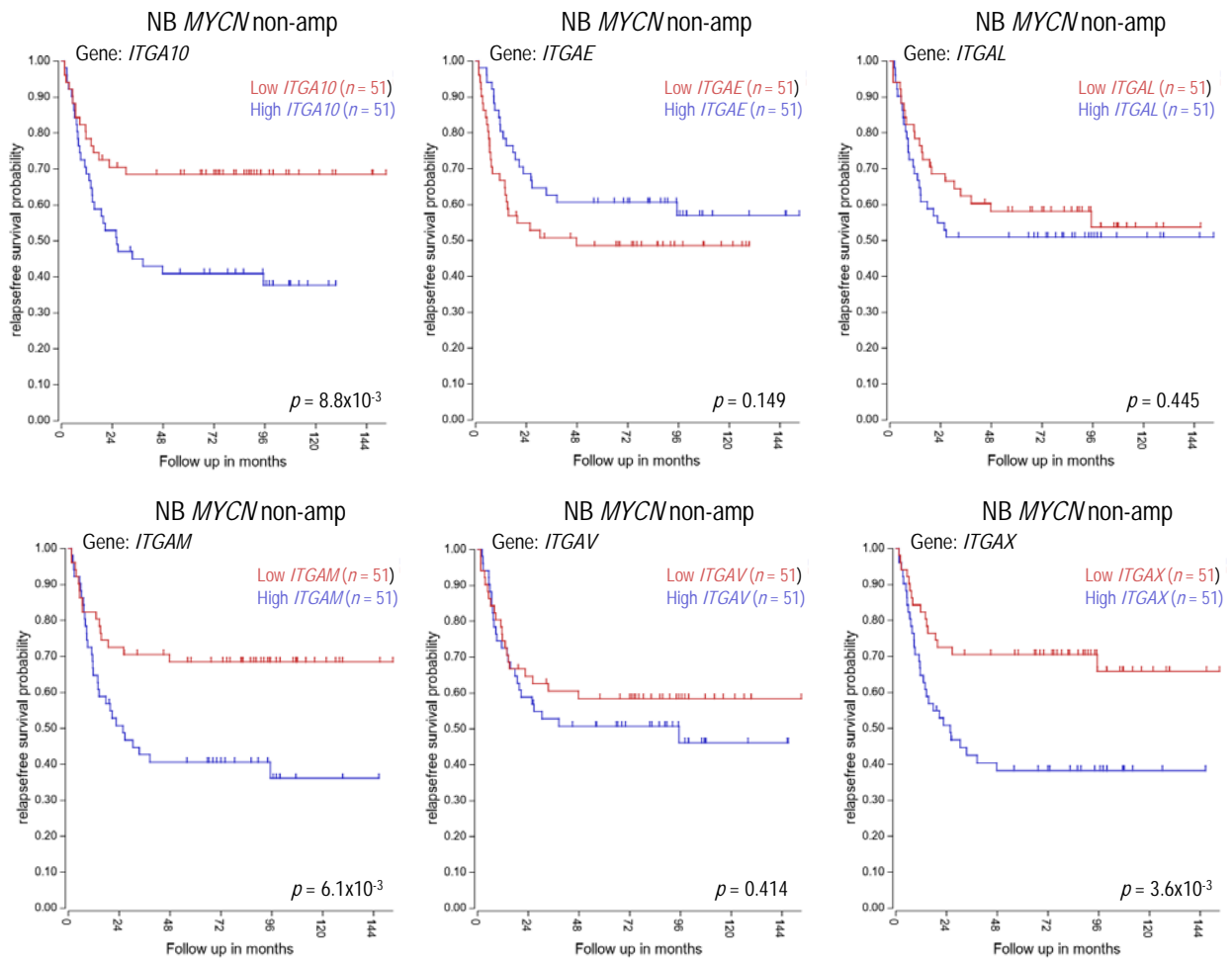

Supplement: Supplementary file 8 — Supplementary file8 (PDF 110 KB) [file 18_2022_4557_MOESM8_ESM.pdf]

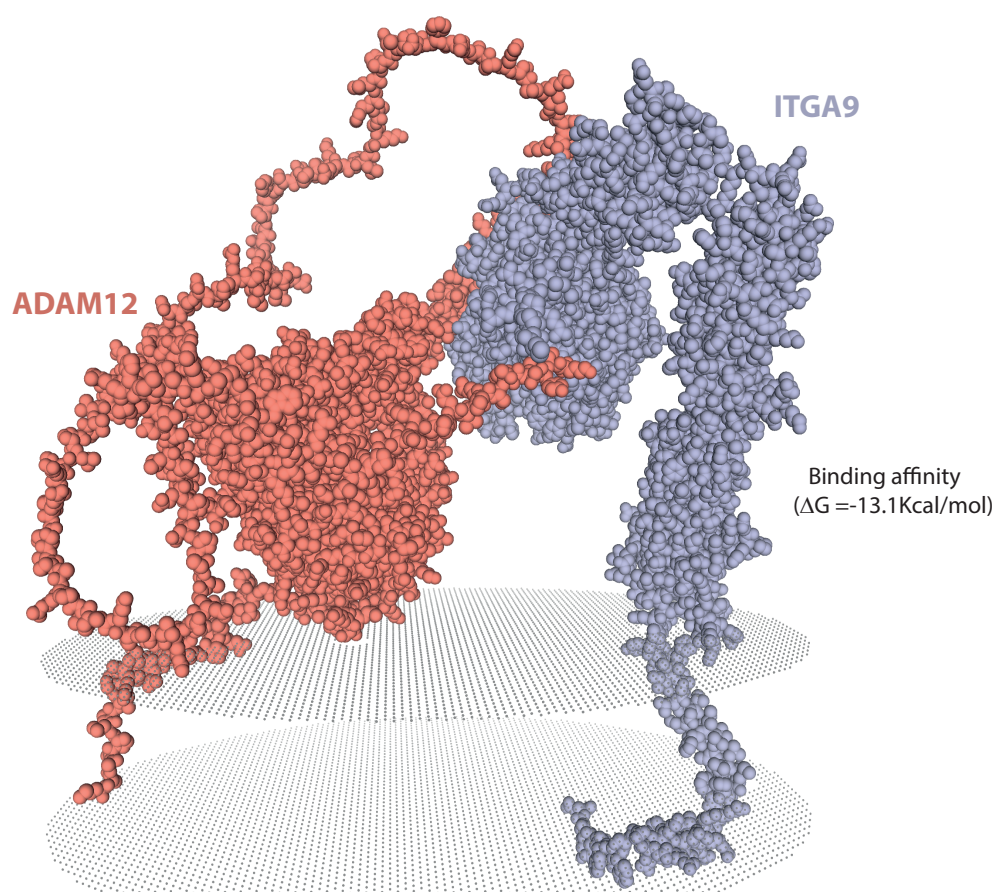

Supplement: Supplementary file 9 — Relapse-free survival curves of NB MYCN non-amplified patients considering each alpha integrin expression. For each alpha integrin analysed, samples (n = 102) were separated in high or low expression (PDF 11810 KB) [file 18_2022_4557_MOESM9_ESM.pdf]

**a**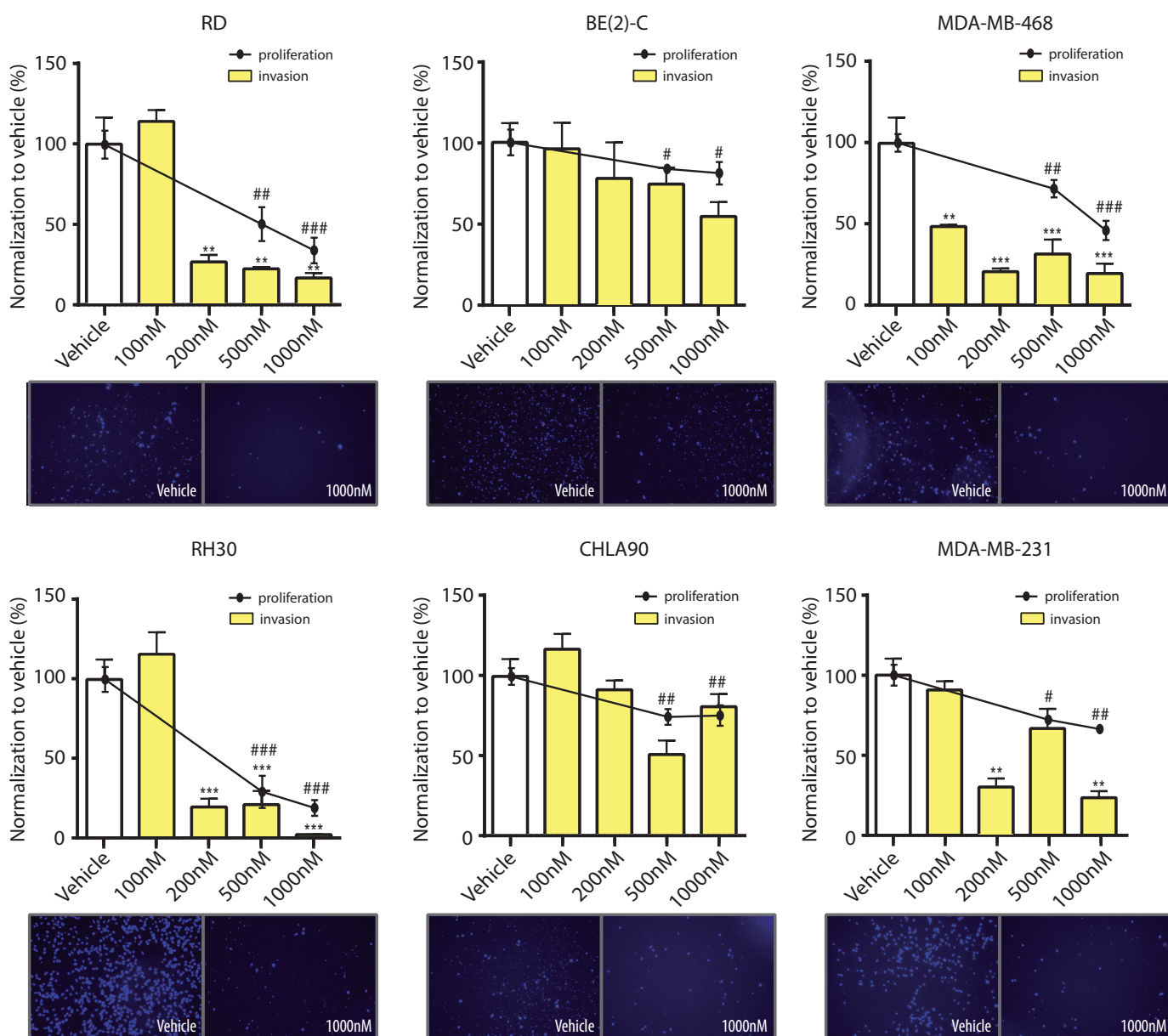**b**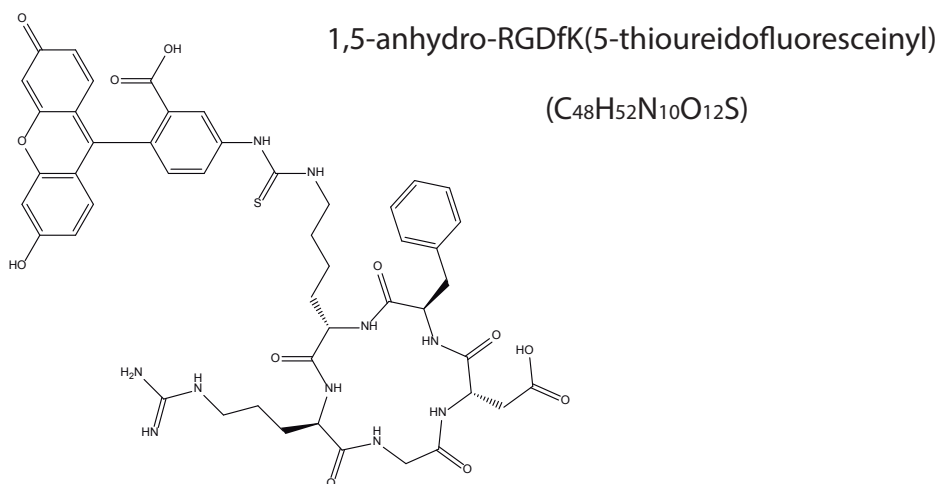

Supplement: Supplementary file 10 — Treatment with RGD peptide significantly reduces cell invasiveness and cell growth. a. Relative cell invasiveness and cell proliferation after treatment with increasing concentrations of RGD peptide (100nM, 200nM, 500nM and 1000nM) in RMS (RD and RH30), NB (BE(2)-C and CHLA90) and BC (MDA-MB-468 and MDA-MB-231). Bars represent cell invasion (significance indicated with asterisks), whereas cell proliferation is represented by dots and lines (significance indicated with hashes). Representative images of the Transwell-invasion assays, including control cells and cells treated with 1000nM are also shown below each plot. All measurements were taken in triplicates. All values are expressed in percentages and referred to those of the control condition. Statistical significance (* or # p<0.05, ** or ## p<0.01, *** or ### p<0.001) was obtained after one-way ANOVA test. b. Molecular structure, name and chemical formula of the RGD peptide used for this experiment (PDF 5713 KB) [file 18_2022_4557_MOESM10_ESM.pdf]
